# Split dosing of artemisinins does not improve antimalarial therapeutic efficacy

**DOI:** 10.1038/s41598-017-12483-4

**Published:** 2017-09-21

**Authors:** N. J. White, J. Watson, E. A. Ashley

**Affiliations:** 1 Mahidol Oxford Tropical Medicine Research Unit, Faculty of Tropical Medicine, Mahidol University, 420/6 Rajvithi Rd, Bangkok, 10400 Thailand; 2 Myanmar Oxford Clinical Research Unit, Yangon, Myanmar; 3 Centre for Tropical Medicine & Global Health, Nuffield Department of Clinical Medicine, University of Oxford, Oxford, UK

## Abstract

It has been suggested recently, based on pharmacokinetic-pharmacodynamic modelling exercises, that twice daily dosing of artemisinins increases malaria parasite killing and so could “dramatically enhance and restore drug effectiveness” in artemisinin resistant *P. falciparum* malaria infections. It was recommended that split dosing should be incorporated into all artemisinin combination regimen designs. To explain why parasite clearance rates were not faster with split dose regimens it was concluded that splenic malaria parasite clearance capacity was readily exceeded, resulting in the accumulation of dead parasites in the circulation, that parasite clearance was therefore an unreliable measure of drug efficacy, and instead that human immunity is the primary determinant of clearance rates. To test these various hypotheses we performed a logistic meta-regression analysis of cure rates from all falciparum malaria treatment trials (n = 40) with monotherapy arms containing artemisinin or a derivative (76 arms). There was no evidence that split dosing enhanced cure rates.

## Introduction

When artemisinin and its derivatives were first evaluated in the treatment of malaria a variety of doses and dosing schedules were assessed. Following single or multiple doses, rates of parasite clearance in falciparum and vivax malaria were faster than observed previously with other classes of antimalarial drug, but satisfactory cure rates with artemisinins alone in falciparum malaria required dosing for more than five days^1–10^. The artemisinins are eliminated rapidly (t_1/2_~1 hour) but giving them twice or even three times in one day did not appear to provide additional benefit over once daily administration, so this became the norm^4–14^. There were a few exceptions. In uncomplicated malaria artemether-lumefantrine required twice daily administration because of the readily saturated oral absorption of lumefantrine. In severe malaria there was concern that in a highly synchronous infection in which mature schizonts predominated, sub-maximal effects might result from the first parenteral administration so a second dose was given at 12 hours as an “insurance policy”.

Recently, based on pharmacokinetic-pharmacodynamic (PK-PD) modelling studies, it has been suggested that twice daily dosing of artemisinins could increase parasite killing^14–17^ and one study went as far as to claim that it could “dramatically enhance and restore drug effectiveness” in artemisinin resistant infections^18^. The authors further recommended “that twice-daily dosing should be incorporated into *all* artemisinin combination treatment (ACT) regimen design considerations as a simple and effective way of ensuring the continued long-term effectiveness of ACTs”. The Liverpool group’s strong recommendation for a major change in dosing, which would have a profound effect on current and future practices if followed, was based on PK-PD modelling and was not supported by clinical trial data^19^. The modelling predicted that splitting current once daily ACT doses into twice per day administration would increase parasite killing enormously (by a factor of 10^8^)^16–18^. So have treatment recommendations been wrong all these years - or is there something wrong with the modelling?

## PK-PD modelling

Standard PK-PD models of antimalarial drug concentration-effect relationships generally parameterise the ‘PD’ component (parasite killing) as a sigmoid –Emax relationship driven by plasma concentrations in which parasite numbers decline as a first-order process for a given drug concentration. From observed 48-hour cycle parasite reduction ratios (PRR) and measured plasma concentration profiles in artemisinin treated patients, these models imply that parasite killing rates are very high for a few hours following drug administration, and then decline rapidly as concentrations of artesunate (or artemether) and their main metabolite dihydroartemisinin all fall. It follows logically from this model construct that the effect of each dose is equivalent (in terms of PRR) when suitably spaced out in time, i.e. if the first dose reduced the number of parasites by a factor of 10^4^ then a dose 6–8 hours later would also reduce numbers by 10^4^ fold resulting in a 10^8^ total reduction. However, similar rates of parasitaemia decline (over 48 hours) are observed whether artemisinins are given once, twice or even three times in a day^19^. Furthermore parasite clearance is actually slower with protracted exposures to the antimalarial peroxides following slowly absorbed intramuscular artemether and slowly eliminated artefenomel than it is with parenteral artesunate which is eliminated very rapidly^19–21^. This may be considered an example of ‘all models are wrong, but some are useful’. The standard PK-PD models are useful for predicting PRR for once daily administration of artemisinin but they fail to predict the observed PRR when more than one dose is given per day. Recent modelling claims to have ‘solved’ this model misfit by hypothesising that the splenic clearance capacity for  infected erythrocytes is exceeded by parasite killing by artemisinin antimalarials^16,17^. It was conjectured that this " saturation" of clearance capacity resulted in the accumulation of dead parasites in the circulation thereby dissociating parasite killing from parasite clearance. Based on this conjecture, it was argued that parasite clearance was an unreliable measure of drug efficacy (as the proportions of live and dead parasites could not be distinguished). Furthermore it was concluded unreservedly that “human immunity is the primary determinant of clearance rates, unless or until artemisinin killing has fallen to near-ineffective levels”^22^ and more recently that “ the impact of human immunity in clearing erythrocytes containing dead or dying parasites makes parasite clearance rates highly insensitive and non-specific diagnostics of resistance”^23^. If indeed these conjectures are all true they would deal a serious blow to current epidemiological assessments of artemisinin resistance, which rely heavily on this metric for phenotyping and for validating the parasite genotyping used in molecular surveillance^19,24–28^. This study examines whether evidence from previous clinical studies of the efficacy of artemisinin and its derivatives supports this hypothesized model structure and the derived therapeutic recommendations.

## Clinical observations

The radical suggestions of the Liverpool group are not supported by clinical observations. If immunity is the primary determinant of parasite clearance rates it cannot explain why parasite clearance rates are twice as slow in patients of similar age and geographic origin, who have K13 mutant artemisinin-resistant compared with K13 wild type artemisinin-sensitive parasites^19,24–28^. In studies where the effects of immunity, or age as a surrogate of cumulative exposure, on parasite clearance rates have been quantitated, the effects are much smaller than the effects of artemisinin resistance^19,24,29,30^. In assessing treatment responses in artemisinin resistant falciparum malaria the hypothesis that human immunity is the *primary* determinant of parasite clearance rate^17^ does not fit the facts.

## Cure rates following single versus split dosing

If parasite killing substantially exceeds splenic clearance capacity, and giving artemisinins twice daily is so much better than once daily as claimed^18^, then irrespective of effects on parasite clearance there should be *substantial* differences in cure rates with twice daily versus once daily administration.

To test this hypothesis we performed a logistic meta-regression analysis of all trials with monotherapy arms containing artemisinin or a derivative. The dependent variable was cure rate, and the independent variables were duration of treatment (in days) and number of artemisinin doses. If the Liverpool group’s hypothesis was correct then the number of doses given should have been a significant covariate. A further meta-regression model was run on all studies of oral artesunate with the mg/kg dose as an independent variable. The logistic meta-regressions were run with a random effect for each study (random intercept term) and fixed effects for the number of doses and duration of treatment (and dose). We fitted the model in *R* (version 3.1.1) using the function *glmer* from the *lme4* package. The database of extracted study meta-data can be found in the supplementary materials.

## Search strategy

We searched the WorldWide Antimalarial Resistance Network (WWARN) Clinical Trials Publication library for eligible studies. This online resource contains all antimalarial clinical efficacy trials conducted and published since 1960^31^. Studies were eligible if once daily or more frequent administration of artemisinin or a derivative was used as monotherapy to treat uncomplicated falciparum malaria and if cure rates at 28 days were reported for each treatment group (PCR adjusted or unadjusted). For two studies the numbers of failures by day 28 were not reported and were requested from the corresponding author.

In early studies where patients were kept in hospital for 28 days to assess cure rate (i.e. reinfection was not possible) the result was combined with later community based studies with PCR adjusted estimates.

## Results

**Figure 1:**
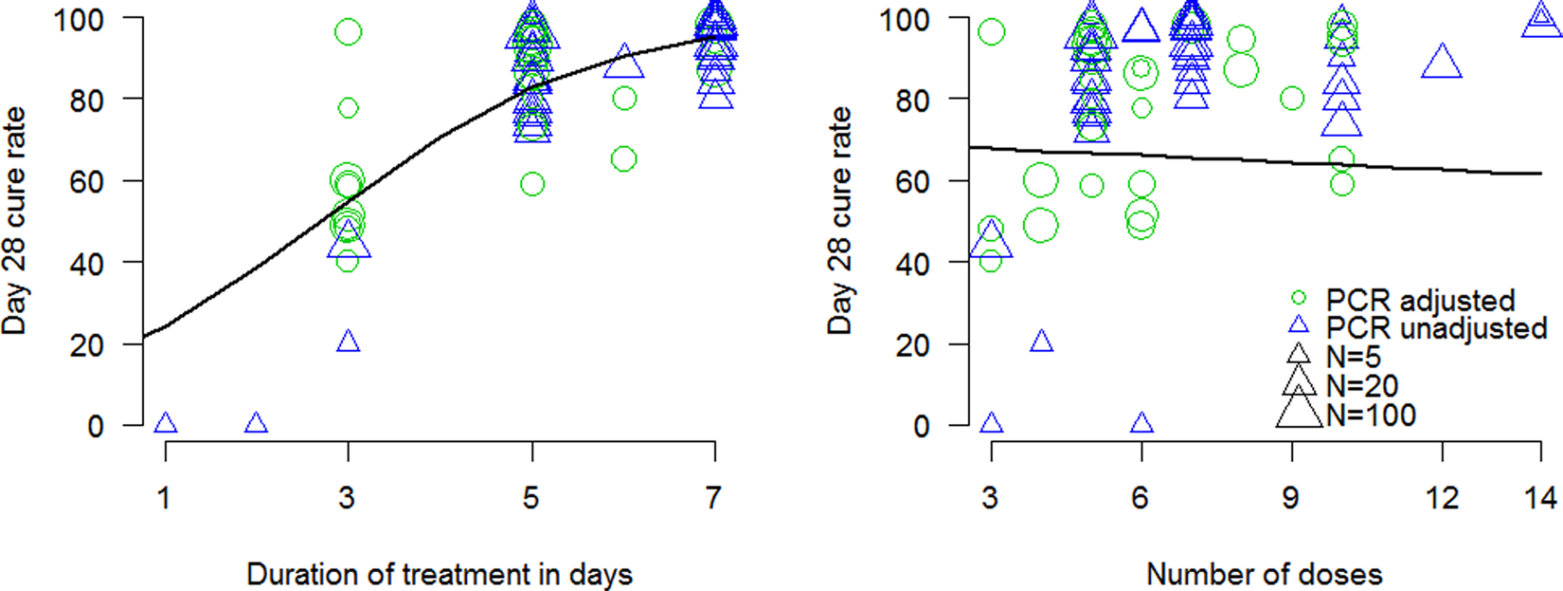
The relationship between duration of treatment and day 28 cure rate (left panel); and number of artemisinin doses and day 28 cure rate (right panel). The marginal model fit for each independent variable is shown by the thick black lines.

The search identified 40 studies, comprising 76 distinct treatment arms, which met the inclusion criteria (37 from the WWARN database and 3 from secondary searches of references in those articles). The earliest study was reported in 1984, and the most recent in 2016. The majority of studies were performed in Asia (n = 33) with seven reported from Sub-Saharan Africa. Twenty-five studies included participants aged 15 years or older, thirteen enrolled children and adults and for two studies this information was not available. The treatment arms were artesunate (n = 48) artemether (10), artemisinin (10), dihydroartemisinin (7) or beta-cyclodextrin-artemisinin complex (1) all given in a variety of doses, dosing intervals, routes and treatment course durations (Fig. 1).

The logistic meta-regression model estimated that the duration of treatment was the main determinant of efficacy (95% confidence interval did not contain zero) and that the number of doses explained none of the residual variance (95% confidence interval was centred approximately at 0). Thus for regimens of artemisinins that were sub-optimal (i.e. gave sub-maximal cure rates), having more than one dose per day provided no benefit over once daily administration. To check the assumption that each dose administered in these various studies produces the maximum parasiticidal effect a logistic meta-regression was performed using only studies with the most frequently evaluated drug, oral artesunate, (n = 39). This found that the dose in mg/kg was not a significant predictor of efficacy (lowest doses were 1.6 mg/kg). This confirms that ~2 mg/kg gives almost maximum antiparasitic effects in artemisinin-sensitive *P. falciparum* infections.

## Discussion

For antimalarial treatments with artemisinin or its derivatives there is no evidence that split dosing either accelerates parasite clearance or augments cure rates significantly. In those studies where cure rates were sub-maximal, and the conjectured “dramatic enhancement of drug effectiveness” should have been evident, none was found. An effect of dose frequency on treatment efficacy was observed in a small study of 43 patients in which once daily artemether-lumefantrine was compared with standard twice daily dosing. In that study PCR adjusted cure rates were 85.1% and 94.4% respectively but this difference was explained entirely by 30% lower lumefantrine levels in the former group since lumefantrine exposure is the principal determinant of cure following treatment with this ACT^32^. In studies of artemisinin monotherapies where twice or thrice daily administration has been evaluated, and in contemporary or sequential comparisons with once daily administration there is no evidence that cure rates are substantially higher with more than once daily dosing^1,3–13,33–38^.

Furthermore there is no evidence that splenic clearance functions are as low as  the values needed to sustain this hypothesis^16,18,39^. Taken together there is no clinical support for these PK-PD modelling predictions, which appear to be wrong. The fundamental problem is probably the modelling of parasite killing and clearance in falciparum malaria as a simple first order process^14,18,39^. Whilst the decline in parasite densities is log linear, and can therefore be described as a first order process, it seems that once daily exposures to artemisinin or its derivatives produce maximum effects in the majority of patients. The kinetics of malaria parasite killing and parasite recovery are more complex than currently modelled.

### Data availability statement

The data are uploaded as a supplementary file.

## Electronic supplementary material

Dataset 1
